# Outcome of cognitive performances in bipolar euthymic patients after a depressive episode: a longitudinal naturalistic study

**DOI:** 10.1186/s12991-015-0070-2

**Published:** 2015-10-13

**Authors:** Ramona Păunescu, Ioana Micluţia

**Affiliations:** Department of Neurosciences, Discipline of Psychiatry and Paediatric Psychiatry, University of Medicine and Pharmacy “Iuliu Hatieganu”, 43 Victor Babes Street, Cluj-Napoca, Romania

**Keywords:** Bipolar disorder, Cognitive functions, Euthymia, Depression

## Abstract

**Background:**

Cognitive functions have been investigated across depressed, manic, hypomanic, mixed and euthymic episodes of bipolar disorder, but the stability or the progression of cognitive impairment is still under research.

**Objective:**

The purpose of the present study was to assess the outcome of cognitive functions in bipolar patients following a depressive episode, after a 6-month period in the absence of mood symptoms.

**Method:**

63 bipolar patients were tested with a battery of neurocognitive tests both at baseline (during an acute depressive episode) and after 6 months of euthymia. The cognitive domains assessed included memory, attention, verbal fluency, processing speed and executive functions. Cognitive performances were compared with those of a control group (40 healthy control subjects), both in depression and in euthymia.

**Results:**

Patients scored worse than control subjects in several cognitive domains, both in depression and euthymia. The most impaired cognitive functions were executive functions and verbal memory. Between the two moments of assessment bipolar patients obtained a significant improvement in memory, verbal fluency, attention and information processing speed. Psychomotor speed showed no difference between depression and euthymia.

**Conclusions:**

Bipolar patients showed impairment in several cognitive domains during depression. A certain degree of impairment remained even after the remission of the affective episode in relationship with the executive functions. Between depression and euthymia, bipolar patients showed important cognitive improvements.

## Background

The interest on cognition related to bipolar disorder noticed a growing interest during the last decade. Cognitive impairments have been outlined in all acute mood episodes of the illness [1–7] but also during euthymic phases [8–11].

Data from the literature show impairment in the majority of cognitive areas (attention, memory, verbal fluency, set shifting) for patients with bipolar disorder. Among these cognitive domains, attention, verbal learning, declarative memory, and executive functions were proposed as trait markers for bipolar disorder [12–15].

Attention deficits were highlighted in several studies; Torres et al. [9] concluded that patients with affective episodes show performance difficulties in areas related to attention which concern visual and motor processing speed, accuracy and reaction time at the tasks of maintaining attention that require identifying targets. Sustained attention or vigilance is impaired in patients with bipolar disorder regardless of whether they are evaluated during episodes of mania or depression, as shown in the study of Najit et al. [16]. Another study [17] demonstrated impairment in attention and response inhibition (prolonged reaction times and decreased discriminability) and also an inability to delay reward within bipolar disorder.

Memory deficits for patients with bipolar disorder consist of verbal memory, working memory and visual memory impairments [11]. Significant impairment in verbal recall and recognition in bipolar depression was outlined by Bearden et al. in 2006 [18]. The study of Altshuler et al. [19] demonstrated lower CVLT (California verbal learning test) recall scores for bipolar patients with a poorer functioning as well as a relationship between the impairment in verbal declarative memory and poor role functioning.

Dixon et al. [20] observed a certain pattern of cognitive dysfunctions in manic, depressed and euthymic bipolar patients in terms of strategic thinking, inhibitory control and response initiation regardless of the affective episode. In a recent review, executive functions, alongside with verbal memory were proposed as potential cognitive endophenotypes for bipolar disorder [21].

The progression of cognitive impairment has been reported by a number of studies [22–24], whereas others stress that cognitive impairment is in a strong relationship with acute illness episodes [25, 26].

## Methods

63 bipolar patients, both genders, with ages between 18 and 60 were included into the study. All patients had a minimum level of 8 years of education. The patients underwent clinical and neurocognitive evaluations twice: first time during an acute depressive episode; the second time after 6 months of euthymia. The inclusion criteria for depressive patients were DSM IV-TR and ICD-10 diagnosis of Bipolar Disorder and Hamilton Depression Rating Scale (HAM-D) >8. For euthymia, inclusion criteria consisted of HAM-D scores <7 and Young Mania Rating Scale (YMRS) scores <6. Exclusion criteria were chronic alcoholism or any other substance dependence, dementia, mental retardation, history of head trauma, or any current medical condition which could interfere with the level of cognitive performances. Patients were compared to 40 healthy control subjects matched by demographic characteristics and who met the same exclusion criteria. The study was approved by the University of Medicine and Pharmacy Iuliu Hatieganu Ethics Committee and all patients signed an informed consent before being admitted in the study.

### Demographic and clinical data

Demographic data (age, gender, education level) and clinical information (number of previous affective episodes, types of previous episodes, history of psychotic symptoms) were collected through the clinical interview and completed with data from medical records. First clinical assessment was performed upon admission to the hospital for a depressive episode. The diagnosis was set according to DSM IV-TR and ICD-10 diagnosis criteria for bipolar disorder and major depressive episode; an additional inclusion criterion was HAM-D score >8. Remission was defined by the absence of any affective symptoms for 6 months and scores on HAM-D <7 and YMRS <6. Depressive patients were recruited while being hospitalized in acute emergency wards; therefore, their episodes and symptoms were more severe than those of an outpatient population. From baseline to follow-up, all bipolar patients were treated with a combination of 2 or 3 medications. Thus, 14 patients were treated with a combination of antipsychotics and mood stabilizers; 26 patients were treated with a combination of antipsychotics and antidepressants; 23 patients underwent treatment with a combination of antipsychotics, mood stabilizers and antidepressants.

### Neurocognitive assessment

Cognitive functions were assessed using the following test batteries: Brief Assessment of Cognition in Schizophrenia (BACS) [27], Trail Making Test A and B (TMT A, TMT B) [28] and Wisconsin Card Sorting Test (WCST)-128 Card Version [29]. Subtests of each battery were used to assess specific cognitive domains as follows:

- Verbal memory-List learning test (BACS)
- Working memory-Digit sequencing test (BACS)
- Attention and processing speed
  - Symbol coding test (BACS)
  - Trail Making Test A
- Verbal fluency-Category instances test (BACS)
- Controlled oral word association test (BACS)
- Motor speed-Token motor test (BACS)
- Executive functions
  - Tower of London (BACS)
  - Trail Making Test B
  - Total correct trials (WCST)
  - Total errors (WCST)
  - Perseverative errors (WCST)
  - Conceptual levels (WCST)
  - Number of categories completed (WCST)

### Statistical analysis

Statistical analysis was performed using IBM Statistical Package for Social Sciences 19 (SPPS) software, Windows version.

## Results

### Demographic and clinical characteristics of the patients and control group

Bipolar patients and healthy control group were matched for all demographic items (gender, age, level of education). Demographic and clinical information is summarized in Table 1.

**Table 1:** Demographic and clinical data of bipolar group (*n* = 63) and control group (*n* = 40)

Demographic and clinical aspects	Bipolar patients (*n* = 63) mean/SD	Normal controls (*n* = 40) mean/SD
Age (in years)	48.87 (SD = 12.037)	44.70 (SD = 11.820)
Sex		
1. Male	*n* = 18 (28.57 %)	*n* = 10 (25 %)
2. Female	*n* = 45 (71.42 %)	*n* = 30 (75 %)
Level of education (years)	11.86 (SD = 3.115)	11.55 (SD = 2.708)
HAM-D scores (depression)	21.17 (SD = 4.324)	–
HAM-D scores (euthymia)	4.4603 (SD 1.9823)	3.15 (SD = 2.248)
YMRS scores (euthymia)	1.936 (SD = 1.5102)	1.4 (SD = 1.1047)

*HAM-D* Hamilton Depression Rating Scale, *YMRS* Young Mania Rating Scale, *SD* standard deviation

### Comparison of cognitive functions between depressed patients and control group

The assessment of differences between cognitive performances of depressed bipolar patients and healthy controls was performed using Independent Samples *t* test. The equality of variances of the two independent groups was analyzed through Levene test. Results are presented in Table 2.

**Table 2:** Comparison of mean scores of cognitive assessments between depressed bipolar patients and controls

Cognitive function	Group	Mean scores	Std. dev	Std. dev error	*F*	Sig.	*t*	Mean difference	*p* value
Memory									
Verbal memory (list learning test)	Patients	6.2381	2.31958	0.29224	0.077	0.0782	−4.214	−2.0119	0.000
	Control	8.2500	2.42709	0.38376					
Working memory (digit Sequencing test)	Patients	15.75	5.995	0.755	0.258	0.612	−1.919	−2.354	0.058
	Control	18.10	6.184	0.978					
Attention and processing speed									
Trail making test A	Patients	57.70	24.878	3.134	3.495	0.064	2.467	10.598	0.15
	Control	47.10	13.608	2.152					
Symbol coding	Patients	35.22	15.419	1.943	0.478	0.491	−2.575	−7.553	0.11
	Control	42.78	12.927	2.044					
Verbal fluency									
Category instances test	Patients	14.67	5.385	0.678	2.474	0.119	−2.259	−2.708	0.026
	Control	17.38	6.709	1.061					
Controlled oral word association test	Patients	12.92	5.589	0.674	5.152	0.025	−3.568	−4.529	0.001
	Control	17.45	7.243	1.126					
Motor speed (token motor test)	Patients	75.59	13.272	1.672	3.002	0.086	−2.798	−7.013	0.006
	Control	82.60	10.862	1.717					
Executive functions									
Tower of London	Patients	10.84	5.976	0.753	0.569	0.425	−3.123	−3.759	0.002
	Control	14.60	5.472	0.865					
Trail making test B	Patients	115.02	53.643	6.758	16.174	0.000	4.974	37.066	0.000
	Control	77.95	19.845	3.138					
WCST-total correct	Patients	75.33	8.910	1.123	0.154	0.695	1.338	21.102	0.184
	Control	72.97	8.402	1.328					
WCST-total errors	Patients	37.62	16.844	2.122	18.452	0.000	6.442	18.744	0.000
	Control	18.88	9.235	1.460					
WCST-preservative errors	Patients	20.57	13.102	1.651	25.235	0.000	5.026	10.946	0.000
	Control	9.63	5.256	0.831					
WCST-conceptual levels	Patients	65.41	6.707	0.845	0.029	0.865	−0.976	−1.287	0.332
	Control	66.70	6.227	0.985					
WCST-categories completed	Patients	5.40	0.814	0.103	169.397	0.000	6.272	9.348	0.000
	Control	6.00	0.000	0.000					

*WCST* Wisconsin Card Sorting Test, *Mean scores* mean scores for each group, *Std. dev* standard deviation, *Std. dev error* standard deviation error, *F F* test, *Sig* significance, *t t* statistic, *mean difference* Difference between group means, *p* value probability

#### Memory

##### Verbal memory

Verbal memory was evaluated with List learning—a subtest within BACS battery. Patients performed worse than controls (mean score 6.231; SD = 2.31958 vs mean score 8.2500; SD = 2.42709), obtaining lower total scores for the five consecutive trials of the cognitive test.

##### Working memory

The mean score for working memory in patients was 15.75 (SD = 5.995) while healthy controls obtained a mean score of 18.10 (SD = 6.184). Even if controls scored higher, the difference between the two groups had a low statistical significance (*p* < 0.1).

#### Attention and information processing speed

Depressed patients and controls obtained similar mean scores both for the time of completion in TMT-A (57.70; SD = 24.878 vs 47.10; SD = 13.608) as well as for the total number of trials in symbol coding test (35.22; SD = 15.419 vs 42.78; SD = 12.927).

#### Verbal fluency

For category instances test, the difference between the two groups was low (*p* = 0.026). For the controlled oral word association test, the mean for the control group was 17.45; SD = 7.423 and the mean for patients was 12.92; SD = 5.589 (*p* = 0.001).

#### Motor speed

The psychomotor speed was tested with the token motor test within BACS. Controls performed better with a mean score of 82.60 (SD = 10.862) while the mean score for patients was 75.89 (SD = 13.272).

#### Executive functions

Executive functions were assessed with several cognitive tests. For Tower of London test within BACS, the difference between patients and controls was significant for the threshold of 5 %. For WCST, we compared raw scores for the total number of correct trials, total errors, total preservative errors, conceptual levels and the number of categories completed. While for the number of total correct trials and number of conceptual levels the two groups performed similarly (*p* = 0.184 and *p* = 0.332), for the total number of errors and preservative errors, as well as for the number of categories completed, patients performed worse than controls (*p* < 0.001). For the B version of TMT, the differences between the two groups were also significant at the threshold of 1 % (*p* < 0.001) both for the time needed to complete the trial and for the total number of errors.

### Comparison of mean scores of bipolar patients between depression and euthymia

We used paired samples *t* test to compare the scores obtained by bipolar patients between the two times of assessment, as presented in Table 3.

**Table 3:** Comparison of mean scores of bipolar patients between depression and euthymia

Cognitive function	Mean	Std. dev	95 % CI		*t*	*p* value
			Lower	Upper		
Memory						
Verbal memory (list learning test)	−1.5619	0.88174	−1.2569	−0.81286	−9.316	0.000
Working Memory (digit sequencing test)	−1.603	3.221	−2.414	−0.792	−3.951	0.000
Attention and processing speed						
Trail making test A	3.968	10.400	1.349	6.587	3.029	0.004
Symbol coding	−3.556	7.291	−5.392	−1.719	−3.871	0.000
Verbal fluency						
Category instances test	−1.921	3.916	−2.097	−0.934	−3.893	0.000
Controlled oral word association test	−1.095	4.169	−2.145	−0.045	−2.085	0.041
Motor speed (token motor test)	−1.333	8.016	−3.352	0.685	−1.320	0.192
Executive functions						
Tower of London	−1.254	3.742	−2.196	−0.312	−2.660	0.010
Trial making test B	9.540	19.384	4.658	14.421	3.906	0.000
WCST-total correct	−0.825	5.695	−2.260	0.609	−1.150	0.254
WCST-total errors	7.794	4.307	6.709	8.878	14.361	0.000
WCST-preservative errors	5.365	4.800	4.156	6.574	8.871	0.000
WCST-conceptual levels	−1.571	6.400	−3.183	0.040	−1.949	0.056
WCST-categories completed	−0.349	0.513	−0.478	−0.220	−5.403	0.000

*WCST* Wisconsin Card Sorting Test, *Std. dev* standard deviation, *CI* confidence interval, *t* *t* statistic, *p value* probability

Results displayed in Table 3 show that bipolar patients obtained significant improvement of cognitive performances between the two moments of assessment (depression and euthymia) for the following domains: verbal memory (Pearson correlation coefficient = 0.933, *p* < 0.001), working memory (Pearson correlation coefficient = 0.854, *p* < 0.001), verbal fluency (category instances subtest-Pearson correlation coefficient = 0.763; *p* < 0.001; controlled oral association—Pearson correlation coefficient = 0.716; *p* < 0.05), attention (Pearson correlation coefficient = 0.909, *p* < 0.05 for TMT-A and Pearson correlation coefficient = 0.887; *p* < 0.001 for symbol coding subtest). Executive functions tested with Tower of London test and TMT-B also improved between the two moments (Pearson correlation coefficient = 0.797; *p* < 0.01 and Pearson correlation coefficient = 0.936; *p* < 0.001). For WCST there were no differences between depression and euthymia for the total number of correct trials and conceptual levels (*p* = 0.254; *p* = 0.056), but the total number of errors and preservative errors, as well as the number of categories completed improved significantly (*p* < 0.001). Psychomotor speed did not show significant changes between the two moments (Pearson correlation coefficient = 0.804; *p* = 0.192).

### Comparison of mean scores of cognitive assessments between euthymic bipolar patients and control

Table 4 shows the differences of cognitive performances obtained by the patients after 6 months of euthymia and healthy controls. The results show similar performances in most cognitive areas. For executive functions assessed with TMT-B, the patients obtained significantly lower scores than controls (*p* < 0.001) and had a higher number of errors (*p* = 0.004). The other executive functions scores were only significant for a threshold of 5 % (*p* = 0.030 for Tower of London subtest = 0.013 for WCST-conceptual levels). Scores obtained by the control group for verbal fluency and motor speed were also higher than those of the patients (*p* = 0.012 for controlled oral association subtest and *p* = 0.017 for token motor test).

**Table 4:** Comparison of mean scores of cognitive assessments between euthymic bipolar patients and controls

Cognitive function	Group	Mean scores	Std. dev	Std. dev error	*F*	Sig.	*t*	Mean difference	*p* value
Memory									
Verbal memory (list learning test)	Patients	7.8000	2.20907	0.27832	0.704	0.404	−0.970	−0.45000	0.335
	Control	8.2500	2.42709	0.38376					
Working memory (digit sequencing test)	Patients	17.35	5.930	0.747	0.350	0.555	−0.616	−0.751	0.539
	Control	18.10	6.184	0.978					
Attention and processing speed									
Trail making test A	Patients	53.73	21.880	2.757	2.985	0.087	1.716	6.630	0.089
	Control	47.10	13.608	2.152					
Symbol coding	Patients	38.78	15.286	1.926	0.165	0.685	−1.371	−3.997	0.173
	Control	42.78	12.927	2.044					
Verbal fluency									
Category instances test	Patients	16.59	5.910	0.745	0.913	0.342	−0.625	−0.788	0.533
	Control	17.38	6.709	1.061					
Controlled oral word association test	Patients	13.45	5.270	0.718	6.926	0.010	−2.569	−3.434	0.012
	Control	16.59	7.126	1.126					
Motor speed (token motor test)	Patients	76.92	12.058	1.519	1.218	0.272	−2.419	−5.679	0.017
	Control	82.60	10.862	1.717					
Executive functions									
Tower of London	Patients	12.10	5.738	0.723	0.445	0.506	−2.198	−2.505	0.030
	Control	14.60	5.472	0.865					
Trail making test B	Patients	105.48	45.932	5.787	10.849	0.001	4.182	27.526	0.000
	Control	77.95	19.845	3.138					
WCST-total correct	Patients	73.75	8.690	1.095	0.044	0.835	0.445	0.771	0.658
	Control	72.97	8.402	1.328					
WCST-total errors	Patients	22.11	12.988	1.636	4.439	0.038	1.476	3.236	0.143
	Control	18.88	9.235	1.460					
WCST-preservative errors	Patients	11.40	7.934	1.000	3.983	0.049	1.248	1.772	0.215
	Control	9.63	5.256	0.831					
WCST-conceptual levels	Patients	66.62	6.015	0.758	0.065	0.799	−0.066	−0.081	0.948
	Control	66.70	6.227	0.985					
WCST-categories completed	Patients	5.90	0.296	0.037	20.630	0.000	2.555	−0.095	0.013
	Control	6.00	0.000	0.000					

*WCST* Wisconsin Card Sorting Test, *Mean scores* mean scores for each group, *Std. dev* standard deviation, *Std.dev error* standard deviation error, *F F* test, *Sig* significance, *t t* statistic, *Mean difference* difference between group means, *p value* probability

## Discussions

Several studies have outlined cognitive impairment in different cognitive domains for bipolar patients. The present study is a longitudinal, naturalistic, 6 months follow-up study intended to asses the changes of cognitive performances in bipolar patients after a depressive episode.

Cognitive functions evaluated during depression revealed impairment in several cognitive areas when the patients were compared to healthy controls. Verbal memory was the most affected function within BACS battery (*p* < 0.001). Patients group, when compared to the controls, recorded a smaller number of words recalled for each of the five verbal memory trials. Also, the patients group did not show a significant progression in the number of words remembered after each try, unlike the control group, which did. Our findings are consistent with those reported by other studies [15, 18, 30]. There were no important differences between the 2 groups for working memory. Differences between controls and patients for verbal fluency tests were reported to be significant for the threshold of 5 %. While verbal fluency was reported as persistently impaired in manic episodes [31], data regarding this cognitive domain in the depressive phase of bipolar disorder are limited. Data from previous studies regarding verbal fluency in bipolar disorder are inconsistent. While some research showed differences in verbal fluency tasks for bipolar patients [32, 33], other found no discrepancy between patients with bipolar disorder and control groups [1]. Psychomotor speed was assessed with Token motor test. Depressive patients performed worse than controls (*p* < 0.05), placing, in total, less tokens and having more tokens placed incorrectly. Both tests used for assessing attention showed no differences between the depressed and control group. For executive functions tests, patient performed worse than controls in all measures of assessment. (*p* < 0.001 for TMT-B and WCST; *p* < 0.05 for Tower of London), displaying thus difficulties in set shifting, task switching, conceptual skills, planning and problem solving.

Repeated measures of cognitive functions after 6 months showed significant differences for almost all cognitive areas. These results may conclude that cognitive impairment could be episode related in bipolar disorder. Both verbal and working memory scores improved significantly for remitted patients, who obtained similar performances when compared with controls (*p* < 0.001). Attention and verbal fluency were also corrected for euthymic patients. Motor speed did not improve between the two assessments, suggesting that for this cognitive area, factors other than depression are involved. The study of Mora et al. [34] who recruited 28 bipolar patients and 26 controls showed the persistence of cognitive impairment in areas such as executive functions, inhibition processing speeds and verbal memory which remained affected in bipolar euthymic patients over 6-year follow-up. After 6-month follow-up, our study found persistent impairment for verbal fluency, processing speed and executive functions in euthymic bipolar patients compared to controls. These results are similar to those of the study conducted by Torrent et al. [35] who compared 71 euthymic bipolar patients with 35 controls and reported persistent impaired executive functions (assessed with WCST and TMT A and B) for bipolar euthymic patients. Our results showed that executive functions such as cognitive flexibility and set shifting assessed with TMT-B improved for euthymic patients but remained impaired, when compared to healthy controls (*p* < 0.001).

## Conclusions

During a depressive episode bipolar patients show impairments in most cognitive areas when compared to a healthy control group. After 6 months free of symptoms, patients display similar results with controls for neurocognitive testing for almost all cognitive functions. Executive functions such as the ability to switch between tasks and cognitive flexibility remain impaired even during euthymia. The results of the present study do not support the progression of cognitive impairment, showing that for the bipolar patients, after 6 months free of symptoms, significant improvement is present in most cognitive domains. Nevertheless, all patients included into the study were under psychiatric medication, for the entire duration of the study; therefore, the effect of treatment on cognition could have not been ruled out.

## Abbreviations

DSM IV TR: Diagnostic and Statistical Manual of Mental Disorders, fourth edition, text revision
ICD-10: International Classification of Diseases 10th revision
BACS: Brief Assessment of Cognition in Schizophrenia
TMT A and B: Trail Making Test A and B
WCST: Wisconsin Card Sorting Test
